# Longer time between anterior cruciate ligament injury and reconstruction is associated with a greater risk of medial meniscus injury

**DOI:** 10.1002/jeo2.70101

**Published:** 2024-12-04

**Authors:** Hao Wang, Zhenning Liu, Baoqiang Li, Hao Wu, Liping Pan, Daojian Zhang, Yongping Cao

**Affiliations:** ^1^ Department of Orthopedics Peking University First Hospital Beijing China; ^2^ Department of Orthopedics, Beijing Chao‐Yang Hospital Capital Medical University Beijing China

**Keywords:** Anterior cruciate ligament, Meniscus, Meniscectomy, Suture, Reconstruction

## Abstract

**Purpose:**

To evaluate the impact of delaying reconstruction following anterior cruciate ligament (ACL) injury on the risk of meniscal injury and subsequent meniscectomy, the study was carried out. This constitutes the first study of its kind to be conducted in China.

**Methods:**

This study collected data on patients who had undergone ACL reconstruction surgery at Peking University First Hospital between 2010 and 2022. Patient's injury details, including the time and cause of ACL injury, surgery date and meniscal injury details, were recorded. Patients were stratified into distinct cohorts based on the duration between injury and reconstruction. Logistic regression analysis was used to evaluate the impact of a delayed reconstruction time on the risk of meniscal injury and meniscectomy.

**Results:**

The study involved patients with an average age of 34.1 ± 11.3 years. Nearly half of the patients (49.74%) had meniscal injuries. Univariate logistic regression analysis showed that gender and the time from ACL trauma to surgery were significantly associated with meniscus injury (*p* < 0.01). Men have a higher risk of meniscus damage than women (*p* < 0.01, odds ratio: 1.94, 95% confidence interval: 1.23–3.05). Patients who had surgery 12 months after injury had a significantly increased risk of meniscus injury compared to those who had surgery within 3 months after injury (*p* < 0.01). The time from ACL injury to operation was significantly correlated with medial meniscus injury (*p* < 0.001). There was no significant correlation between time to ACL reconstruction and lateral meniscus injury (*p* > 0.05). Age was significantly associated with the risk of medial meniscectomy (*p* > 0.05). Time from injury to surgery was not significantly associated with the risk of lateral meniscectomy (*p* > 0.05).

**Conclusions:**

Delayed reconstruction beyond 12 months after ACL rupture increases the risk of medial meniscus injury. The risk of meniscus injury after ACL injury is higher in men than in women, and the risk of medial meniscectomy increases with age.

**Level of Evidence:**

Level III.

**Abbreviations**
ACL: anterior cruciate ligament
BMI: body mass index
CI: confidence interval
OR: odds ratio
SD: standard deviation

## INTRODUCTION

The anterior cruciate ligament (ACL) is an important structure for maintaining the stability of the knee joint, and it is also the most vulnerable structure when the knee joint is injured by professional sports, leisure sports and other reasons [23]. With the increase in life expectancy and the popularity of fitness activities, the rate of ACL injury is increasing [2, 20]. Anatomic ACL reconstruction is the best method to restore the stability of knees with ACL loss [24].

There is still a lack of consensus on the timing of ACL reconstruction. Previous researchers have found that premature ACL reconstruction may increase the risk of postoperative fibrosis and affect the recovery of knee motion after surgery [16, 25]. However, if reconstruction is not carried out in a timely manner, the internal sheer force of knee joints with complete rupture of the ACL increases significantly, leading to possible articular cartilage and meniscus damage [7, 8]. ACL tears with meniscal injury may have a lower healing potential than ACL tears alone [4].

Meniscectomy and suture repair are two common treatments for meniscal tears. Meniscal repair has been shown to produce better clinical outcomes and cause less knee degeneration than meniscectomy [21]. However, the relationship between the time of ACL reconstruction and the risk of meniscus injury and repair remains unclear.

This study represents the first retrospective, single‐centre investigation conducted in the general population of China. Its aim was to examine the impact of a delayed reconstruction time on the risk of meniscal injury and meniscectomy following ACL injury.

## METHODS

### Study design and population

A total of 378 patients who underwent ACL reconstruction at Peking University First Hospital from 2010 to 2022 were enrolled. Each patient had positive clinical tests (anterior drawer test, jerk test and/or Lachman test) and imaging evidence of ACL injury, which was confirmed by arthroscopy during surgery. We excluded patients with multiple ligament injuries, those who underwent previous realignment surgery and those who underwent meniscal or cartilage surgery.

### Data collection

The patients' characteristic data were recorded, including age, sex, height, weight, time of ACL injury, cause of ACL injury, limb side of ACL injury, surgery date of ACL reconstruction, time and location of meniscal injury and surgical method of meniscus repair. The time from injury to surgery and body mass index (BMI) were calculated from these data. The time to ACL reconstruction was calculated as the difference in months between the date of injury and the date of surgery. Patients were divided into several groups according to the time between injury and surgery: 0–3, 3–6, 6–12, 12–24, 24–60 and >60 months. Patients were also divided into groups according to age at surgery: <30 years, 31–50 years and >50 years. We used the World Health Organization's BMI classification criteria to divide BMI into four levels: underweight (BMI < 18.5 kg/m^2^), normal weight (BMI of 18.5–24.9 kg/m^2^), overweight (BMI of 25.0–29.9 kg/m^2^) and obese (BMI ≥ 30.0 kg/m^2^) [29].

Meniscal injuries were divided into medial and lateral meniscal injuries, and surgical procedures for each type of meniscal injury included meniscal suture and meniscectomy. The operative principle was to preserve as much of the functioning meniscus as possible.

### Statistical analysis

Statistical descriptions were performed using numbers and percentages, means and standard deviations or medians and quartiles, depending on the type of data.

To explore the relationship between the presence of meniscal lesions and the time between injury and surgery, age, sex, BMI, cause of injury and injured limb side were analyzed by univariate logistic regression analysis, where variables were considered continuous and categorical. For variables with *p* < 0.01, the relationship between the presence of meniscal lesions and independent variables was explored by multivariate logistic regression. Similarly, the possible associations of meniscal surgery with these variables were explored by univariate logistic regression analysis, where variables were treated as continuous and categorical variables.

A *p *< 0.05 was considered significant. All analyses and calculations were performed using SPSS 25.

## RESULTS

### Demographic data

A total of 378 patients, including 110 males and 268 females with an average age of 34.1 ± 11.3 years, were included in this study (Table 1). The mean BMI for all patients was 25.5 ± 3.8 kg/m^2^. The time from injury to surgery was divided into six categories: within 3 months, accounting for 61.11% of patients; 3–6 months, accounting for 9.52% of patients; 6–12 months and 12–24 months, each accounting for 6.88% of patients; 24–60 months, accounting for 7.67% of patients and over 60 months, accounting for 7.94% of patients.

**Table 1:** Patient demographic data.

Variable	Included patients (*n* = 378)	
	Mean ± SD	Median (range)
Age (years)	34.1 ± 11.3	33 (14–64)
<30	155 (41.01%)	
30–50	179 (47.35%)	
>50	44 (11.64%)	
Sex		
Male	110 (29.1%)	
Female	268 (70.9%)	
BMI (kg/m^2^)	25.5 ± 3.8	25.2 (17.4–40.1)
Normal weight	179 (47.35%)	
Underweight	8 (2.12%)	
Overweight	139 (36.77%)	
Obese	52 (13.76%)	
Time from trauma‐surgery (months)	14.3 ± 36.9	1 (1–312)
0–3	231 (61.11%)	
3–6	36 (9.52%)	
6–12	26 (6.88%)	
12–24	26 (6.88%)	
24–60	29 (7.67%)	
>60	30 (7.94%)	
Cause of injury		
Football	30 (7.94%)	
Badminton	9 (2.38%)	
Basketball	45 (11.9%)	
Other sports	56 (14.81%)	
Trauma	238 (62.96%)	
Limb side of injury		
Left	194 (51.32%)	
Right	184 (48.68%)	
Meniscal lesion	188 (49.74%)	
Meniscal lesion location		
Medial	112 (29.63%)	
Lateral	102 (26.98%)	
Medial meniscal lesion treatment		
Suture	62 (55.36%)	
Meniscectomy	50 (44.64%)	
Lateral meniscal lesion treatment		
Suture	36 (35.30%)	
Meniscectomy	66 (64.71%)	

Abbreviations: BMI, body mass index; SD, standard deviation.

Among patients with ACL injuries, 49.74% also suffered from meniscal injuries. Specifically, 45.7% had isolated medial meniscus injuries, 40.4% had isolated lateral meniscus injuries and 13.8% had both medial and lateral meniscus injuries. Among the included patients, there were a total of 112 cases of medial meniscus injuries and 102 cases of lateral meniscus injuries, accounting for 29.63% and 26.98%, respectively.

The treatment of meniscal injuries involves either meniscal repair or meniscectomy. Among all patients with medial meniscal injuries, meniscal repair accounted for 55.36%, and meniscectomy accounted for 44.64%. For patients with lateral meniscal injuries, meniscal repair accounted for 35.30%, while meniscectomy accounted for 64.71%.

### Logistic regression analysis of meniscal injury

Univariate logistic regression analysis of meniscal injury showed that sex and the time from trauma to surgery were significantly associated with meniscal injury (*p* < 0.01) (Table 2). The risk of meniscal injury in males was higher than that in females (*p* < 0.01, odds ratio [OR]: 1.94, 95% confidence interval [CI]: 1.23–3.05). Comparing patients who underwent surgery within 3 months after injury with patients who underwent surgery 12 months after injury, the risk of meniscal injury was significantly increased (*p* < 0.01), but there was no significant difference in the risk of meniscal injury in patients who underwent surgery within 3–6 months and 6–12 months (*p* > 0.05). After adjusting for sex, the time from trauma to surgery was still significantly associated with meniscal injury (*p* < 0.01). Compared to patients who underwent surgery within 3 months of injury, those who underwent surgery 12–24 months, 24–60 months and >60 months after injury had 2.44 times (*p* < 0.05, OR: 2.44, 95% CI: 1.03–5.77), 3.26 times (*p* < 0.01, OR: 3.26, 95% CI: 1.37–7.72) and 3.09 times (*p* < 0.01, OR: 3.09, 95% CI: 1.34–7.13) higher risks of developing meniscal injury, respectively. However, the risk of meniscal injury was not significantly different between patients who underwent surgery within 3 months and those who underwent surgery between 3 and 12 months after injury (*p* > 0.05).

**Table 2:** Univariate logistic regression analysis of meniscal lesions.

Variables	Meniscal lesions		OR (95% CI)	*p* Value
	No	Yes		
Age (years)	34.86 ± 11.28	33.27 ± 11.29		
<30	85 (45.21%)	70 (36.84%)	1	0.23
30–50	84 (44.68%)	95 (50%)	0.73 (0.47–1.12)	0.15
>50	19 (10.11%)	25 (13.16%)	0.63 (0.32–1.23)	0.17
Sex				
Female	68 (35.79%)	42 (22.34%)	1	
Male	122 (64.21%)	146 (77.66%)	1.94 (1.23–3.05)	<0.01*
BMI (kg/m^2^)	25.31 ± 4.11	25.74 ± 3.48		
Normal weight	94 (49.47%)	85 (45.21%)	1	0.68
Underweight	5 (2.63%)	3 (1.6%)	0.66 (0.15–2.86)	0.58
Overweight	65 (34.21%)	74 (39.36%)	1.26 (0.81–1.96)	0.31
Obese	26 (13.68%)	26 (13.83%)	1.11 (0.6–2.05)	0.75
Time from trauma‐surgery (months)	7.62 ± 19.05	20.98 ± 47.81		
0–3	127 (66.84%)	104 (55.32%)	1	<0.01*
3–6	20 (10.53%)	16 (8.51%)	0.98 (0.48–1.98)	0.95
6–12	17 (8.95%)	9 (4.79%)	0.65 (0.28–1.51)	0.31
12–24	9 (4.74%)	17 (9.04%)	2.31 (0.99–5.39)	0.05*
24–60	8 (4.21%)	21 (11.17%)	3.21 (1.36–7.53)	0.01*
>60	9 (4.74%)	21 (11.17%)	2.85 (1.25–6.49)	0.01*
Cause of injury				
Soccer	12 (6.32%)	18 (9.57%)	1	0.37
Badminton	3 (1.58%)	6 (3.19%)	1.33 (0.28–6.39)	0.72
Basketball	19 (10%)	26 (13.83%)	0.91 (0.36–2.34)	0.85
Other sports	30 (15.79%)	26 (13.83%)	0.58 (0.24–1.42)	0.23
Trauma	126 (66.32%)	112 (59.57%)	0.59 (0.27–1.28)	0.19
Limb side of injury				
Left	103 (54.21%)	91 (48.4%)	1	
Right	87 (45.79%)	97 (51.6%)	1.26 (0.84–1.89)	0.26

Abbreviations: BMI, body mass index; CI, confidence interval; OR, odds ratio.

* *p* < 0.05 indicates a significant difference.

Univariate logistic regression analysis of medial meniscus injury showed that the time from injury to operation was significantly related to medial meniscus injury (*p *< 0.001), while age, sex, BMI, cause of injury and limb side of injury were not significantly related (*p *> 0.05) (Table 3). Compared with that of patients who underwent surgery within 3 months after injury, the risks of medial meniscal injury were 3.53 times (*p* < 0.001, OR: 3.53, 95% CI: 1.54–8.09), 3.78 times (*p* < 0.001, OR: 3.78, 95% CI: 1.71–8.35) and 3.53 times (*p* < 0.001, OR: 3.53, 95% CI: 1.62–7.70) higher for patients who underwent surgery 12–24 months, 24–60 months and more than 60 months after injury, respectively. The risk of medial meniscus injury was not significantly different between patients who underwent surgery within 3 months and those who underwent surgery 3–12 months after injury (*p* > 0.05).

**Table 3:** Univariate logistic regression analysis of medial meniscal lesions.

Variables	Medial meniscal lesions		OR (95% CI)	*p* Value
	No	Yes		
Age (years)	34.05 ± 11.32	34.13 ± 11.31		
<30	108 (40.6%)	47 (41.96%)	1	0.78
30–50	125 (46.99%)	54 (48.21%)	0.99 (0.62–1.59)	0.98
>50	33 (12.41%)	11 (9.82%)	0.77 (0.36–1.64)	0.49
Sex				
Female	84 (31.58%)	26 (23.21%)	1	
Male	182 (68.42%)	86 (76.79%)	1.53 (0.92–2.54)	0.1
BMI (kg/m^2^)	25.26 ± 3.91	26.13 ± 3.49		
Normal weight	132 (49.62%)	47 (41.96%)	1	0.34
Underweight	7 (2.63%)	1 (0.89%)	0.40 (0.05–3.35)	0.4
Overweight	92 (34.59%)	47 (41.96%)	1.44 (0.88–2.33)	0.14
Obese	35 (13.16%)	17 (15.18%)	1.36 (0.70–2.66)	0.36
Time from trauma‐surgery (months)	9.56 ± 27.77	25.45 ± 50.98		
0–3	180 (67.7%)	51 (45.5%)	1	<0.001*
3–6	26 (9.8%)	10 (8.9%)	1.36 (0.61–3.00)	0.45
6–12	18 (6.8%)	8 (7.1%)	1.57 (0.65–3.82)	0.32
12–24	13 (4.9%)	13 (11.6%)	3.53 (1.54–8.09)	<0.001*
24–60	14 (5.3%)	15 (13.4%)	3.78 (1.71–8.35)	<0.001*
>60	15 (5.6%)	15 (13.4%)	3.53 (1.62–7.70)	<0.001*
Cause of injury				
Soccer	17 (6.39%)	13 (11.61%)	1	0.22
Badminton	5 (1.88%)	4 (3.57%)	1.05 (0.23–4.69)	0.95
Basketball	29 (10.9%)	16 (14.29%)	0.72 (0.28–1.86)	0.5
Other sports	43 (16.17%)	13 (11.61%)	0.4 (0.15–1.02)	0.06
Trauma	172 (64.66%)	66 (58.93%)	0.5 (0.23–1.09)	0.08
Limb side of injury				
Left	139 (52.26%)	55 (49.11%)	1	
Right	127 (47.74%)	57 (50.89%)	1.13 (0.73–1.76)	0.58

Abbreviations: BMI, body mass index; CI, confidence interval; OR, odds ratio.

* *p* < 0.05 indicates a significant difference.

Univariate logistic regression analysis of lateral meniscus injury showed that the time from injury to operation, age, sex, BMI, cause of injury and limb side of injury were not significantly related to lateral meniscus injury (*p *> 0.05) (Table 4).

**Table 4:** Univariate logistic regression analysis of lateral meniscal lesions.

Variables	Lateral meniscal lesions		OR (95% CI)	*p* Value
	No	Yes		
Age (years)	34.58 ± 11.37	32.7 ± 11.06		
<30	108 (39.13%)	47 (46.08%)	1	0.45
30–50	134 (48.55%)	45 (44.12%)	0.77 (0.48–1.25)	0.29
>50	34 (12.32%)	10 (9.8%)	0.68 (0.31–1.48)	0.33
Sex				
Female	86 (31.16%)	24 (23.53%)	1	
Male	190 (68.84%)	78 (76.47%)	1.47 (0.87–2.48)	0.15
BMI (kg/m^2^)	25.53 ± 3.94	25.50 ± 3.44		
Normal weight	129 (46.74%)	50 (49.02%)	1	0.98
Underweight	6 (2.17%)	2 (1.96%)	0.86 (0.17–4.4)	0.86
Overweight	102 (36.96%)	37 (36.27%)	0.94 (0.57–1.54)	0.79
Obese	39 (14.13%)	13 (12.75%)	0.86 (0.42–1.75)	0.68
Time from trauma‐surgery (months)	12.65 ± 28.62	18.63 ± 53.11		
0–3	166 (60.14%)	65 (63.73%)	1	0.88
3–6	28 (10.14%)	8 (7.84%)	0.73 (0.32–1.68)	0.46
6–12	21 (7.61%)	5 (4.9%)	0.61 (0.22–1.68)	0.34
12–24	18 (6.52%)	8 (7.84%)	1.14 (0.47–2.74)	0.78
24–60	22 (7.97%)	7 (6.86%)	0.81 (0.33–1.99)	0.65
>60	21 (7.61%)	9 (8.82%)	1.1 (0.48–2.52)	0.83
Cause of injury				
Soccer	23 (8.33%)	7 (6.86%)	0(0‐0)	0.81
Badminton	6 (2.17%)	3 (2.94%)	1.64 (0.32–8.33)	0.55
Basketball	30 (10.87%)	15 (14.71%)	1.64 (0.58–4.69)	0.35
Other sports	40 (14.49%)	16 (15.69%)	1.31 (0.47–3.67)	0.6
Trauma	177 (64.13%)	61 (59.8%)	1.13 (0.46–2.77)	0.79
Limb side of injury				
Left	149 (53.99%)	45 (44.12%)	1	
Right	127 (46.01%)	57 (55.88%)	1.49 (0.94–2.35)	0.09

Abbreviations: BMI, body mass index; CI, confidence interval; OR, odds ratio.

*﻿﻿*p* < 0.05 indicates a significant difference.

### Logistic regression analysis of the risk of meniscectomy

Univariate logistic regression analysis showed that age was significantly associated with the risk of medial meniscectomy (*p *< 0.05). There was no significant relationship between sex, BMI, the time from injury to surgery and the treatment of medial meniscus tears (*p *> 0.05) (Table 5). Compared with patients aged <30 years, patients aged between 30 and 50 years and 50 years or older had a higher risk of meniscectomy. Univariate logistic regression analysis showed that age, sex, BMI and the time from injury to surgery were not significantly associated with the risk of lateral meniscectomy (*p *> 0.05) (Table 6).

**Table 5:** Univariate logistic regression analysis of surgical treatment of medial meniscus lesions.

Variables	Surgical treatment of medial meniscus lesions		OR (95% CI)	*p* Value
	Meniscal suture	Meniscectomy		
Age (years)	30.1 ± 8.92	39.12 ± 12.03		
<30	36 (58.06%)	11 (22%)	1	<0.001*
30–50	25 (40.32%)	29 (58%)	3.8 (1.6–8.98)	<0.001*
>50	1 (1.61%)	10 (20%)	32.73 (3.76–284.83)	<0.001*
Sex				
Female	12 (19.35%)	14 (28%)	1	
Male	50 (80.65%)	36 (72%)	0.62 (0.26–1.49)	0.28
BMI (kg/m^2^)	25.78 ± 3.72	26.57 ± 3.16		
Normal weight	29 (46.77%)	18 (36%)	1	0.67
Underweight	1 (1.61%)	0 (0%)	0 (0–0)	1
Overweight	23 (37.1%)	24 (48%)	1.68 (0.74–3.82)	0.21
Obese	9 (14.52%)	8 (16%)	1.43 (0.47–4.39)	0.53
Time from trauma‐surgery (months)	19.11 ± 49.81	33.3 ± 51.83		
0–3	34 (54.84%)	17 (34%)	1	0.15
3–6	4 (6.45%)	6 (12%)	3 (0.75–12.08)	0.12
6–12	4 (6.45%)	4 (8%)	2 (0.44–8.99)	0.37
12–24	7 (11.29%)	6 (12%)	1.71 (0.5–5.9)	0.39
24–60	9 (14.52%)	6 (12%)	1.33 (0.41–4.36)	0.63
>60	4 (6.45%)	11 (22%)	5.5 (1.52–19.86)	0.01
Cause of injury				
Soccer	7 (11.29%)	6 (12%)	1	0.09
Badminton	3 (4.84%)	1 (2%)	0.39 (0.03–4.8)	0.46
Basketball	11 (17.74%)	5 (10%)	0.53 (0.12–2.42)	0.41
Other sports	11 (17.74%)	2 (4%)	0.21 (0.03–1.36)	0.1
Trauma	30 (48.39%)	36 (72%)	1.4 (0.43–4.62)	0.58
Limb side of injury				
Left	29 (46.77%)	26 (52%)	1	
Right	33 (53.23%)	24 (48%)	0.81 (0.39–1.71)	0.58

Abbreviations: BMI, body mass index; CI, confidence interval; OR, odds ratio.

*p < 0.05 indicates a significant difference.

**Table 6:** Univariate logistic regression analysis of surgical treatment of lateral meniscus lesions.

Variables	Surgical treatment of lateral meniscus lesions		OR (95% CI)	*p* Value
	Meniscal suture	Meniscectomy		
Age (years)	31.14 ± 10.38	33.55 ± 11.4		
<30	19 (52.78%)	28 (42.42%)	1	0.61
30–50	14 (38.89%)	31 (46.97%)	1.5 (0.64–3.55)	0.35
>50	3 (8.33%)	7 (10.61%)	1.58 (0.36–6.9)	0.54
Sex				
Female	12 (33.33%)	12 (18.18%)	1	
Male	24 (66.67%)	54 (81.82%)	2.25 (0.89–5.72)	0.09
BMI (kg/m^2^)	25.29 ± 3.95	25.61 ± 3.15		
Normal weight	16 (44.44%)	34 (51.52%)	1	0.78
Underweight	1 (2.78%)	1 (1.52%)	0.47 (0.03–8.01)	0.6
Overweight	13 (36.11%)	24 (36.36%)	0.87 (0.35–2.14)	0.76
Obese	6 (16.67%)	7 (10.61%)	0.55 (0.16–1.9)	0.34
Time from trauma‐surgery (months)	13.08 ± 41.84	21.65 ± 58.43		
0–3	25 (69.44%)	40 (60.61%)	1	0.74
3–6	3 (8.33%)	5 (7.58%)	1.04 (0.23–4.74)	0.96
6–12	2 (5.56%)	3 (4.55%)	0.94 (0.15–6.01)	0.95
12–24	1 (2.78%)	7 (10.61%)	4.38 (0.51–37.71)	0.18
24–60	3 (8.33%)	4 (6.06%)	0.83 (0.17–4.04)	0.82
>60	2 (5.56%)	7 (10.61%)	2.19 (0.42–11.38)	0.35
Cause of injury				
Soccer	1 (2.78%)	6 (9.09%)	1	0.74
Badminton	0 (0%)	3 (4.55%)	/	1
Basketball	7 (19.44%)	8 (12.12%)	0.19 (0.02–1.99)	0.17
Other sports	6 (16.67%)	10 (15.15%)	0.28 (0.03–2.9)	0.29
Trauma	22 (61.11%)	39 (59.09%)	0.3 (0.03–2.62)	0.27
Limb side of injury				
Left	18 (50%)	27 (40.91%)	1	
Right	18 (50%)	39 (59.09%)	1.44 (0.64–3.27)	0.38

Abbreviations: BMI, body mass index; CI, confidence interval; OR, odds ratio.

^*^ ﻿﻿*p* < 0.05 indicates a significant difference.

## DISCUSSION

The incidence of meniscal injury after ACL injury is nearly 50% in the general population of China, and the incidence of medial and lateral meniscal injury is similar. In our study cohort, the risk of secondary medial meniscus injury began to increase significantly after 12 months. In addition, in our study, the risk of lateral meniscal injury was not significantly related to the time from injury to surgery (*p* > 0.05). Our findings are consistent with those of some foreign studies, which also found a significant increase in the risk of medial meniscus injury 12 months after ACL injury, while the risk of lateral meniscal injury did not change significantly [8, 27]. In addition, our results are similar to, but different from, some recently published studies. Giordano et al., who studied women, found that the risk of secondary medial meniscus injury was significantly increased only in the group who underwent surgery at 24–60 months and >60 months compared with the group who underwent surgery within 0–3 months (*p* < 0.05) [11]. James and Prodromidis et al. found that delaying ACL reconstruction for 3 months significantly increased the risk of meniscal injury, especially medial meniscal injury [12, 22]. In these studies, the time points at which the risk of meniscal injury increased significantly were different, which may reflect that the risk of secondary knee meniscal injury did not increase uniformly at different stages after ACL injury.

This nonlinear variation in meniscal injury risk may be related to a number of factors. After ACL injury, patients may be involved in different exercise levels and rehabilitation processes at different time periods, which will cause differences in the amount of activity and load on the knee joint, thus affecting the meniscal injury. For example, in the early stages after an ACL injury, patients may actively avoid excessive activity and load to reduce stress on the joint. In addition, they may be undergoing conservative treatment or rehabilitation. However, in later stages, patients may gradually increase the amount of activity and load or have abandoned conservative treatment or rehabilitation training, which may increase the risk of meniscus damage. A randomized controlled study of ACL reconstruction at different time points after injury would be ideal, but there are practical and ethical difficulties in conducting such a trial. Future large‐scale prospective studies can be performed for further verification.

Most studies have demonstrated a significant correlation between medial meniscal injury and delayed surgical intervention after injury, although the specific time points at which the risk of meniscal injury increased varied across studies. In contrast, no such association was found for lateral meniscal injury. We hypothesized that an important reason for this difference is the anatomical difference between the medial and lateral menisci. The medial meniscus rim is tightly connected to the joint capsule and is continuous and tightly fixed to the deep part of the medial collateral ligament, while the lateral meniscus rim does not have a continuous attachment, and its attachment is partially interrupted at the popliteus tendon hiatus and the lateral collateral ligament. Therefore, after ACL injury, the medial meniscus is more susceptible to continuous or intermittent crushing and tearing due to poor mobility than the lateral meniscus, which plays a major role in limiting tibial advancement. It has been demonstrated that anterior tibial translation increases by 7–13 mm with ACL loss, and the resultant force on the medial meniscus doubles at flexion angles of 30–90°, resulting in higher stresses within the medial meniscus [19]. The medial meniscus is an important limiting factor in ACL‐deficient knees, acting as a buffer for tibial advancement [13, 28]. In contrast, the lateral meniscus is less restrictive. Some studies have found that lateral meniscal injury does not cause significant knee instability in the absence [30] of ACL; a study of cadaveric knees showed that lateral meniscectomy had no significant effect on anterior tibial translations in ACL‐deficient knees [18].

Our study found a higher risk of meniscal injury after ACL injury in men than in women (*p* < 0.05). The results are consistent with those of some studies, which may be due to differences in injury mechanisms or activity between men and women [2, 14, 27]. Our study found no statistically significant relationship between BMI and medial or lateral meniscal injury. The results are similar to those of some studies. Cristiani et al. found no significant association between BMI and either medial or lateral meniscal injury [8]. Giordano et al. found no statistically significant medial meniscus damage in overweight patients compared to normal‐weight and low‐weight patients [11]. Theoretically, the higher the BMI, the greater the compression of the meniscus and the higher the chance of damage [1, 31]. The effect of high and low BMI on the meniscus may not be significant in the context of the generally limited activity level of patients following ACL injury. We believe that high and low BMI represent different pathophysiological states in patients, which not only affect their risk of injury to the motor system but also reflect or influence their lifestyle habits, which may buffer the risk of injury. For example, in theory, a patient with a larger BMI has a greater weight bearing on the knee joint, which is more likely to cause meniscus wear; however, a patient with a higher BMI may have a low activity habit throughout their lives and may have a greater awareness of protecting their motor system, which may reduce their chances of meniscus wear. Therefore, a high BMI does not necessarily increase the risk of meniscal injury.

Meniscal surgery includes partial or total meniscectomy, meniscal tear repair and open or arthroscopic surgery. An important surgical principle in meniscectomy is to preserve as much of the functioning meniscus as possible [9, 17]. Regeneration following meniscectomy presents significant challenges and may result in an elevated prevalence of osteoarthritis [5]. In addition, the absence of the meniscus also increases the in situ force of the ACL replacement graft, resulting in a higher risk of graft failure [19]. Arthroscopic surgeons are working to develop a range of meniscal repair techniques to address tears of varying complexities and locations. In addition, rapid advances in cell biology and tissue engineering may further improve the healing rate of injuries traditionally considered irreparable [3, 9].

This study found no significant relationship between the time from injury to reconstruction and the risk of medial or lateral meniscectomy. This is similar to the results of Giordano et al. [11], who divided the delay in surgery after injury into several time periods and found that the time from injury to reconstruction did not increase the risk of meniscectomy. However, the results of this study also differ from those of some studies. For example, Giordano et al. divided the time period into <12 months and >12 months and found that a delay of more than 12 months after injury increased the risk of nonrestoration of the medial meniscus. Similarly, Krutsch et al. found that delaying ACL reconstruction for 6 months significantly increased the risk of meniscectomy [15]. In addition, the study of Chhadia AM et al. found that the increased risk of medial meniscus injury and the decreased repair rate were closely related to the increased operation time [6]. Giordano and Krutsch et al., who simply divided all patients into two groups according to the surgical delay time after injury, did not consider the possible large differences in activity and load of patients at different time periods. Theoretically, if the time period is divided carefully enough, the amount of knee joint activity and load of a patient for a fixed time period is relatively constant. Therefore, we preferred to divide the time period carefully to better control this confounding factor. In addition, the differences in these studies may be related to regional differences, population differences and sample size differences. One study found that medial meniscus repair rates in young athletes decreased by 7% per month from ACL injury to reconstruction [26]. However, there is a large difference in the level of exercise between young athletes and ordinary patients, the amount of exercise and load on the knee joint is quite different and injury to the medial meniscus may also be very different. Therefore, this study found no significant relationship between the risk of meniscectomy and the delay time to reconstruction in the general population. Meniscus surgery depends on the location, type and size of the injury. Delayed reconstruction time after ACL injury, while increasing the risk of meniscal injury, does not appear to exacerbate the extent of the injury or increase the risk of resection. This may be related to the quality of each patient's meniscus and their inherent living habits and exercise level.

This study found that age was significantly associated with the risk of medial meniscectomy (*p* < 0.05), with a higher risk of meniscectomy in the <30–50‐ and >50‐year‐old age groups than in the <30‐year‐old age group. Studies have found that the meniscus undergoes significant degeneration over a person's lifetime, and the incidence of meniscal tears increases with age [10]. This has led surgeons to prefer resection to suturing in elderly patients.

This study is the first large‐scale retrospective study on the relationship between the time interval between ACL injury and reconstruction and medial meniscus injury in the general population in China. The results of the study reflect the living habits and exercise level of the general population in China to a certain extent. In addition to careful grouping of reconstruction time intervals, we controlled for confounders such as age, sex, BMI, cause of injury and injured limb side, and our sample was heterogeneous with respect to these variables, which makes our results more generalizable. This study has great reference significance for the timing of surgery after ACL injury in the general population of nonprofessional athletes, especially in the Chinese population.

This was a single‐centre retrospective study, and its results have some limitations, mainly reflected in selection bias and confounding bias. The study included patients who underwent surgery after being admitted through outpatient care, which depended on each patient's personal preference and the surgeon's treatment beliefs. Information about patients who declined surgery or were never treated was lacking. In addition, there were some uncontrollable confounding factors in this study, including the postinjury knee joint activity and load, as well as the conservative treatment and rehabilitation training course that each patient may have undergone. Although the postinjury time was divided in detail to reduce the impact of this factor, it was still difficult to adjust for this confounding factor using objective data. In future large‐scale prospective studies, corresponding measures can be taken to monitor and measure patients' joint activity load and related treatments.

## CONCLUSION

In the Chinese general population, delayed reconstruction more than 12 months after ACL rupture increases the risk of medial meniscus injury, but does not increase the risk of meniscectomy. There was no significant correlation between ACL injury and operative time and lateral meniscus injury. Age was significantly associated with the risk of medial meniscectomy. The time from injury to surgery was not significantly associated with the risk of lateral meniscectomy. As people live longer and become more physically active, the likelihood of ACL injury increases. Optimizing the reconstruction time, reducing the risk of meniscus injury and ensuring successful recovery after surgery can significantly improve the quality of life of patients.

## AUTHOR CONTRIBUTIONS

Hao Wang and Yongping Cao devised the study. Hao Wang and Baoqiang Li performed the measurements. Hao Wang and Zhenning Liu did the analysis. Hao Wang wrote the first draft. Hao Wu and Liping Pan revised it. Daojian Zhang and Yongping Cao did the final revision of the manuscript. All authors have read and approved the final version of the manuscript.

## CONFLICT OF INTEREST STATEMENT

The authors declare no conflicts of interest.

## ETHICS STATEMENT

This retrospective chart review study involving human participants was in accordance with the ethical standards of the institutional and national research committee and with the 1964 Helsinki Declaration and its later amendments or comparable ethical standards. So, the study was approved by the Ethics Committee of Peking University First Hospital（NO. 2023‐160). Informed consent to participate in the study was taken from patients. As this study is retrospective, informed consent is not required with the approval of the Ethics Committee.

## Data Availability

The raw data sets are available from the corresponding author upon reasonable request.
